# Supplementary material for: Efficacy and Safety of Chemotherapy Combined with Hormonal Therapy in Heavily Pretreated Advanced Epithelial Ovarian, Fallopian Tube, or Primary Peritoneal Cancer (ELSA/KGOG3049): A Multicenter Pilot Study
Source: Cancers (Basel). 2025 Jul 12;17(14):2320. doi: 10.3390/cancers17142320 (PMC12293222; doi:10.3390/cancers17142320)
Supplement: Supplementary file 1 [file cancers-17-02320-s001.zip › cancers-3724313-supplementary.pdf]

Table S1. Adverse events.

	Grade 1	Grade 2	Grade 3
Case 1	0	0	0
Case 2	0	0	0
Case 3	0	0	0
Case 4	0	0	0
Case 5	0	0	0
Case 6	0	0	0
Case 7	0	0	0
Case 8	0	0	0
Case 9	0	0	0
Case 10	0	0	0
Case 11	0	0	0
Case 12	0	0	0
Case 13	0	0	0
Case 14	0	0	0
Case 15	0	0	0
Case 16	0	0	0
Case 17	0	0	0
Case 18	0	0	0
Case 19	0	0	0
Case 20	0	0	0
Case 21	0	0	0
Case 22	0	0	0
Case 23	0	0	0
Case 24	0	0	0
Case 25	0	0	0
Case 26	0	0	0
Case 27	0	0	0
Case 28	0	0	0
Case 29	0	0	0
Case 30	0	0	0
Case 31	0	Nausea (possible)	0
Case 32	0	0	0
Case 33	0	0	0
Case 34	0	0	0
Case 35	0	0	0
Case 36	0	0	0
